# Integrating Transcranial Ultrasound Stimulation with Exercise-Based Rehabilitation for Ischemic Stroke Recovery: A Preclinical Study

**DOI:** 10.3390/ijms27198498

**Published:** 2026-09-23

**Authors:** Chang-Mu Chen, Ting-Yu Chang, Lin-Hwa Long, Cheng-Tien Wu, Shing-Hwa Liu

**Affiliations:** 1 Division of Neurosurgery, Department of Surgery, College of Medicine and Hospital, National Taiwan University, Taipei 10051, Taiwan; cmchen10@ntuh.gov.tw (C.-M.C.); linhua1976@yahoo.com.tw (L.-H.L.); 2 Institute of Toxicology, College of Medicine, National Taiwan University, Taipei 10051, Taiwan; d03447003@ntu.edu.tw; 3 Department of Nutrition, China Medical University, Taichung 406040, Taiwan; 4 Department of Medical Research, China Medical University Hospital, China Medical University, Taichung 406040, Taiwan; 5 Department of Pediatrics, College of Medicine and Hospital, National Taiwan University, Taipei 10051, Taiwan

**Keywords:** ischemic stroke, low-intensity pulsed ultrasound, apoptosis, BDNF, irisin

## Abstract

Ischemic stroke remains a leading cause of long-term disability worldwide, and effective neurorehabilitative strategies to enhance functional recovery are limited. This preclinical study compared noninvasive low-intensity pulsed ultrasound (LIPUS), aerobic exercise (AE) rehabilitation, and their combination in a mouse model of ischemic stroke. Transient focal cerebral ischemia was induced in male C57BL/6 mice using middle cerebral artery occlusion (MCAO). Male mice assigned to LIPUS-containing groups received LIPUS preconditioning for three consecutive days before MCAO, followed by two days of recovery and 14 days of post-stroke LIPUS, running wheel-based AE, or combined treatment. Functional recovery was assessed using open-field activity, neurological deficit scoring, and rotarod performance. Histological, immunohistochemical, TUNEL, and Western blot analyses were performed. MCAO caused marked motor and neurological impairments and brain tissue injury. LIPUS, AE, and their combination improved functional and histopathological outcomes, with the combined treatment showing greater effects for selected outcomes. All three interventions increased FNDC5 and BDNF and reduced TUNEL positivity. LIPUS and combined treatment, but not AE alone, increased SOD2 and catalase and reduced 8-OHdG immunoreactivity. These findings provide proof-of-concept in male mice to support further investigation of LIPUS combined with exercise rehabilitation for ischemic stroke recovery.

## 1. Introduction

According to the statistics from the World Stroke Organization–Global Stroke Fact Sheet 2025, stroke is currently the second leading cause of death globally, after ischemic heart disease, causing about 7 million deaths annually, and the third leading cause of death and disability combined worldwide [1]. It has been estimated that about a quarter of people aged 25 and older worldwide will suffer a stroke in their lifetime [2]. This has a significant impact on public health. About one-third of these patients require long-term care or rehabilitation. Although stroke represents a chronic and long-term disease with rising incidence, resources dedicated to stroke research remain significantly lower than those allotted to cancer, coronary heart disease, or dementia [1,2,3].

Stroke is broadly classified into ischemic and hemorrhagic types, with ischemic stroke accounting for 70–80% of all cases [3]. Regardless of etiology, neurons exposed to ischemia or hypoxia undergo energy failure, excitotoxicity, calcium overload, oxidative stress, inflammatory cytokine production, matrix metalloproteinase activation, autophagy, endoplasmic reticulum stress, and apoptosis, all contributing to neuronal and glial dysfunction [4,5]. Currently, the primary acute treatment for stroke is thrombolysis with recombinant tissue-type plasminogen activator (rt–PA) or surgical clot removal [1,2]. Given the limited regenerative capacity of the CNS and the high likelihood of permanent impairment, elucidating molecular pathways involved in stroke and identifying effective strategies for neuronal protection or prevention remain urgent and essential research priorities.

Physical exercise is now widely recognized as a positive therapeutic strategy for neurological disorders, including stroke and post-stroke rehabilitation [6]. Animal models demonstrate that exercise promotes neuroprotection by increasing neuronal survival, reducing deficits, limiting blood–brain barrier dysfunction, and improving neurovascular integrity [6,7].

However, inappropriate exercise intensity, either excessively strenuous or overly mild, may fail to produce benefits and may even worsen brain injury. For example, Li et al. reported increased neuronal apoptosis in rats receiving overly intense aerobic training during rehabilitation after ischemic stroke [8]; Svensson et al. observed that strenuous exercise induced stress and neural injury in mice, without reducing stroke risk [9]. Schmidt et al. emphasized that the optimal exercise parameters (e.g., exercise intensity, dose–response correlation) for stroke prevention and rehabilitation remain unclear and require further data from human and animal studies [10].

Ultrasound is commonly used for diagnostic imaging and as a real-time guide for minimally invasive procedures. It is safe, noninvasive, convenient, and relatively low cost. Ultrasound is also a method of treating diseases, such as low-intensity pulsed ultrasound (LIPUS) with intensity < 3 W/cm^2^ and medium frequency range (0.7–3.0 MHz) [11]. Several LIPUS devices have been approved by the US FDA to treat nonunion fractures (e.g., Exogen 2000^®^, in 2000) or to aid fracture healing (e.g., AccelStim Ultrasonic Bone Growth Stimulator, in 2022). Numerous studies also report that LIPUS facilitates neuronal differentiation [12], enhances neural recovery and regeneration [13], and may benefit ischemic stroke recovery [14]. Furthermore, ultrasound techniques have been investigated for thrombolysis in acute ischemic stroke patients [15]. Given its neuroprotective and myoregenerative potential, we propose LIPUS as a supplementary approach to enhance post-stroke exercise rehabilitation and explore its feasibility as an adjunct therapy.

The incidence of ischemic stroke continues to rise. Delayed treatment increases irreversible neuronal injury and results in motor and cognitive impairments. Identifying new strategies to mitigate neural damage is therefore an urgent medical priority. This study is a preclinical trial comparing and testing the efficacy of noninvasive LIPUS, aerobic exercise rehabilitation, or a combination of both in improving functional recovery and neuroprotection following ischemic stroke induced by middle cerebral artery occlusion (MCAO) in male mice. This one LIPUS intensity and one aerobic exercise protocol provide a proof-of-concept based on experimental outcome and preliminary mechanism exploration to determine whether more definitive dose- or intensity-dependent effects and in-depth mechanistic studies are warranted.

## 2. Results

### 2.1. Effects of LIPUS, Aerobic Exercise Rehabilitation, and Combination of Both on Post-Stroke Locomotor Performance and Neurological Outcomes in Mice

We first applied low-intensity pulsed ultrasound (LIPUS) at 100 mW/cm^2^ intensity with a 50% duty cycle to the target cerebrovascular region for 20 min per day over three consecutive days prior to middle cerebral artery occlusion (MCAO). A workflow diagram was shown in Figure 1A. Prior to MCAO, all mice underwent running wheel familiarization three times per week for two weeks. Mice assigned to the LIPUS-containing groups additionally received LIPUS preconditioning for three consecutive days immediately before MCAO. After MCAO surgery, mice were allowed to recover for two days. Beginning on postoperative day 3, mice received LIPUS treatment, aerobic exercise rehabilitation, or their combination for 14 days. Behavioral assessments were performed 24 h after the final intervention, followed by euthanasia and collection of blood and brain tissues. Therefore, the LIPUS protocol used in this study included both a preconditioning phase before MCAO and a post-stroke treatment phase. MCAO-induced stroke mice exhibited significant alterations in locomotor activity and neurological function compared with the sham group (Figure 1B). LIPUS or aerobic exercise rehabilitation did not significantly affect locomotor performance or neurological outcomes in sham-operated mice (Figure 1B). In MCAO mice, LIPUS, aerobic exercise rehabilitation, and their combination attenuated behavioral impairments. The combined treatment produced significantly greater improvements in neurological deficit scores (Figure 1(Bd)) and rotarod performance (Figure 1(Be)) than aerobic exercise alone (& *p* < 0.05, MCAO + E vs. MCAO + E + L).

### 2.2. LIPUS, Aerobic Exercise Rehabilitation, and Combination of Both Attenuate Post-Stroke Neuropathological Alterations

Using the same treatment protocol, we further examined cerebral infarction and histopathological outcomes. H&E staining revealed marked histopathological changes in MCAO-induced stroke mice, including tissue damage in brain regions vulnerable to ischemic injury, such as the hippocampus and cerebral cortex (Figure 2A). TTC staining was used to visualize infarcted brain regions. Representative images suggested smaller infarcted areas in the treatment groups than in the untreated MCAO group; however, infarct volume was not quantitatively assessed in the present study (Figure 2B). LIPUS treatment at 100 mW/cm^2^ intensity, aerobic exercise rehabilitation, and their combination attenuated histopathological alterations in MCAO mice (Figure 2A). Semi-quantitative pathological scoring further showed treatment-associated reductions in neuronal necrosis and neuronal cell loss, hemorrhage, and gliosis (Figure 2C–E). The effects were particularly evident for neuronal necrosis and neuronal cell loss (Figure 2C). Aerobic exercise rehabilitation and the combined treatment also significantly reduced hemorrhage scores compared with the untreated MCAO group (Figure 2D).

To further characterize treatment-associated molecular changes, we examined protein levels of fibronectin type III domain containing 5 (FNDC5) (the precursor of irisin, which is a muscle-derived exercise hormone), brain-derived neurotrophic factor (BDNF), and the antioxidant enzymes SOD2 and catalase. Western blot analysis revealed that LIPUS, aerobic exercise rehabilitation, and their combination significantly increased FNDC5 and BDNF protein expression levels in the brains of MCAO-induced stroke mice (Figure 3). The combined treatment produced greater increases in these protein expression levels for selected comparisons. LIPUS and the combined treatment, but not exercise alone, significantly increased SOD2 and catalase protein expression levels (Figure 3). Consistent with the Western blotting results, FNDC5 expression was significantly reduced in the hippocampus, including the dentate gyrus, of MCAO mice. This reduction was markedly reversed by LIPUS, aerobic exercise, and particularly by the combined treatment (Figure 4). Similarly, BDNF expression was decreased following MCAO and was significantly restored by all three interventions, with the combined-treatment group showing the highest BDNF-positive area and a significantly greater effect than LIPUS alone (Figure 5). In addition, 8-hydroxy-2′-deoxyguanosine (8-OHdG), a marker of oxidative DNA damage, was markedly increased in the hippocampus of MCAO mice. LIPUS and the combined treatment significantly reduced the 8-OHdG-positive area compared with the untreated MCAO group, whereas aerobic exercise alone did not produce a statistically significant reduction. The combined treatment resulted in a significantly lower 8-OHdG-positive area than LIPUS alone (Figure 6). Furthermore, TUNEL staining showed a marked increase in TUNEL-positive cells in the hippocampus of MCAO mice. LIPUS, aerobic exercise, and their combination significantly reduced TUNEL positivity compared with the untreated MCAO group, with the combined treatment producing a greater reduction than aerobic exercise alone (Figure 7). Collectively, these data indicate that the improvements associated with LIPUS and aerobic exercise rehabilitation were accompanied by changes in neurotrophic and antioxidant proteins and reductions in oxidative DNA damage and apoptosis-associated DNA fragmentation.

## 3. Discussion

In the present study, we found that noninvasive LIPUS administration in male mice with MCAO-induced ischemic stroke attenuated pathological damage in the cerebral cortex and hippocampus and reduced TUNEL positivity. The combination of LIPUS and aerobic exercise rehabilitation produced greater effects than individual treatments for selected outcomes, although superiority was not consistently observed across all evaluated endpoints. These functional and histopathological improvements were accompanied by changes in FNDC5, BDNF, and antioxidant enzyme expression, as well as reduced oxidative DNA damage. These findings provide a proof-of-concept in male mice to support further investigation of LIPUS combined with exercise rehabilitation for ischemic stroke recovery.

Physical exercise enhances stress resilience through adrenal and corticosteroid pathways, improves balance and muscle strength, reduces inflammation via glial modulation, and enhances sensory and cognitive functions following stroke [6]. A meta-analysis by Austin et al. examined 47 animal studies (from 4250 titles) and found that moderate-intensity aerobic exercise initiated 24–48 h after stroke reduced lesion volume, oxidative injury, inflammation, apoptosis, and improved vascular permeability, with high relevance to clinical design [16]. A systematic review of 112 human clinical studies indicated that structured aerobic training benefits stroke rehabilitation, with fewer than 10% of participants experiencing arrhythmia [17]. A recent review article showed that aerobic exercise possessed the potential to mitigate post-stroke cognitive impairment in ischemic stroke patients through mechanisms including cerebral blood flow improvement, BDNF-mediated pathway enhancement, neuroinflammation inhibition, and oxidative stress suppression [18]. Consistent with these previous findings, the present study showed that aerobic exercise rehabilitation improved behavioral and neuropathological outcomes in ischemic stroke mice. The combined intervention produced greater improvements in selected outcomes than either LIPUS or exercise alone, although this pattern was not observed consistently across all endpoints.

FNDC5 is a membrane-associated precursor protein that can undergo proteolytic cleavage to generate irisin, which is released into the extracellular space and can act on distant tissues [19]. Interestingly, irisin, a muscle-derived exercise hormone, is an important mediator of exercise benefits. Exercise increases circulating irisin levels, enhances fat metabolism by promoting the browning of white adipose tissue, and improves overall energy expenditure and muscle strength [20]. Irisin production is associated with PPAR–γ coactivator–1 α (PGC1–α) activation. PGC1–α could regulate downstream proteins, such as IL–15, FNDC5, VEGFβ, Lrg1, and TIMP4 in muscle tissues; among these, only FNDC5 has been shown to induce beige adipocyte differentiation [21]. FNDC5 has also been shown to be highly expressed in the brain tissues [22,23]. Wrann et al. have demonstrated that a PGC–1α–FNDC5/irisin pathway contributes to the exercise-induced BDNF expression in the hippocampus [23]. Growing evidence indicates that irisin plays key roles in metabolic disorders, such as diabetes [24] and neurological conditions including Alzheimer’s disease [25]. Yet many mechanistic aspects remain unresolved. Song et al. have recently suggested that LIPUS possesses neuroprotective benefits in a dementia mouse model via increased hippocampal Fndc5/irisin levels [26]. In the present study, we found that aerobic exercise rehabilitation in post-stroke mice significantly increased the protein expression levels of FNDC5 and BDNF in the brains; the combined LIPUS and aerobic exercise intervention produced greater increases in these markers for selected comparisons. The newly added immunohistochemical analyses further demonstrated treatment-associated restoration of FNDC5 and BDNF immunoreactivity in the hippocampus, including the dentate gyrus, consistent with the Western blot findings. In addition, LIPUS and the combined intervention significantly reduced 8-OHdG immunoreactivity, suggesting an association between these interventions and reduced oxidative DNA damage, whereas aerobic exercise alone did not produce a significant reduction in 8-OHdG.

Notably, aerobic exercise alone significantly increased FNDC5 and BDNF but did not significantly increase SOD2 or catalase or reduce 8-OHdG under the present experimental conditions. These findings are not necessarily contradictory, because neurotrophic and antioxidant responses may differ in their sensitivity, time course, and dependence on exercise intensity or duration. The 14-day exercise protocol and single terminal sampling time point may have been sufficient to detect changes in FNDC5 and BDNF but not significant changes in the selected antioxidant markers. In contrast, LIPUS-containing interventions produced significant changes in both neurotrophic and selected oxidative-stress-related markers. However, because the relevant pathways were not directly manipulated, these differences should be interpreted as treatment-associated responses rather than evidence of distinct causal mechanisms. In addition, the absence of significant changes in SOD2, catalase, and 8-OHdG after exercise alone does not exclude other antioxidant or anti-inflammatory adaptations that were not examined in the present study.

Research on the biological effects of high-frequency mechanical waves (high-intensity focused ultrasound; HIFU) dates back to 1917, but technological advances in recent decades have led to a rapid increase in its biomedical applications [27]. Unlike high-intensity focused ultrasound (HIFU; with an intensity of exposure ≥ 3 W/cm^2^), which relies on thermal effects to raise tissue temperature to 42–45 °C for 30–60 min and induce protein denaturation or tumor ablation [28], LIPUS (with an intensity of exposure < 3 W/cm^2^) primarily exerts mechanical and biological effects with significantly lower tissue damage, making it suitable for broader therapeutic use [13,29]. Most ultrasound devices operate at 1–3 MHz, whereas LIPUS commonly uses 1.5 MHz, which offers favorable penetration and reflection properties for deep tissues [13]. The output intensity of LIPUS is typically effective within the 30–1000 mW/cm^2^ range [29]. A recent clinical trial using transcranial ultrasound stimulation (1 MHz, 500 mW/cm^2^) for treating Alzheimer’s disease observed a potential benefit for preserving cognitive function [30]. Su et al. have recently found that LIPUS treatment (1 MHz, 528 mW/cm^2^) in an amyloid precursor protein transgenic Alzheimer’s disease mouse model effectively increases amyloid–β clearance and synaptic integrity, improving cognitive function [31]. LIPUS with 1 MHz and 528 mW/cm^2^ conditions has also been used to prevent ischemic stroke in a mouse model [14]. In the present study, LIPUS stimulation at 1 MHz and 100 mW/cm^2^ was associated with improved neurological and neuropathological outcomes under the combined pre- and post-MCAO intervention paradigm. However, because the LIPUS regimen included both three days of pre-MCAO preconditioning and post-stroke treatment, these findings should be interpreted within a combined preconditioning and post-stroke intervention paradigm rather than as evidence of a purely rehabilitative effect. These findings suggest that LIPUS has the potential to improve neurological outcomes under the present pre- and post-MCAO intervention paradigm and that combining it with aerobic exercise may provide greater benefits for selected rehabilitation outcomes.

The brain consists primarily of neurons and various glial cells. Neurons transmit signals to and from the spinal cord, whereas glial cells, far more abundant, maintain homeostasis, nourish neurons, remove debris, facilitate growth, and modulate synaptic transmission. Astrocytes become reactive (astrogliosis) after stroke and release inflammatory mediators that contribute to neural injury [32]. Microglia, the resident immune cells of the CNS, clear debris and respond to invading pathogens while regulating neuroglial interactions through trophic factors and cytokines, thereby influencing post-stroke pathology [33]. Reactive microglial accumulation has been observed in the region of hippocampal CA1 under a global cerebral ischemia condition in animal models [34]. Physical exercise activity has been shown to possess beneficial effects on regulating microglial activity in healthy and diseased animal models [35]. In an MCAO–induced ischemic stroke rat model, treadmill exercise has been found to attenuate the inflammatory microenvironment in the brain via downregulation of MMP12 in microglia [36]. Moreover, a recent study showed that LIPUS effectively inhibited microglial activation and proinflammatory cytokine release in vitro, and reduced neuroinflammation and enhanced synaptic and neuronal function recovery in a traumatic brain injury mouse model through a modulation of microglial polarization [16]. In the present study, exercise, LIPUS, and their combination improved neurological abnormalities caused by ischemic stroke, with the combined intervention showing greater effects for selected outcomes. However, microglial activation, polarization, and inflammatory mediators were not directly evaluated in the present study. Based on previous studies, modulation of microglial polarization may represent one potential mechanism underlying these effects, but this hypothesis remains to be experimentally verified. Therefore, neuroinflammatory modulation should be considered a hypothesis for future investigation rather than a mechanism demonstrated by the present data.

### Limitations

The present study has several limitations: (1) Because the LIPUS treatment was initiated 3 days before MCAO, a preconditioning effect before ischemic injury cannot be excluded. Accordingly, the observed benefits cannot be interpreted solely as post-stroke rehabilitative effects. Rather, the findings should be considered within a combined pre- and post-stroke intervention paradigm. Future studies with pre-treatment-only, post-treatment-only, and combined treatment groups are needed to distinguish preventive neuroprotection from rehabilitative or therapeutic effects. (2) The molecular analyses were based mainly on protein expression and histological observations and therefore do not demonstrate causality. Although the immunohistochemical analyses provide regional support for the observed changes in FNDC5, BDNF, and 8-OHdG, they do not establish causal relationships with functional recovery. Further studies using pathway-specific inhibitors, pharmacological blockade, and, where appropriate, genetic loss-of-function approaches are required to determine the mechanistic roles of FNDC5/irisin, BDNF, antioxidant enzymes, and related signaling pathways. (3) Other important processes involved in post-stroke recovery, including neuroinflammation (such as Iba1 and GFAP, or other pro-inflammatory cytokines), neurovascular regeneration/remodeling, and synaptic plasticity, were not directly examined in the present study. Future studies incorporating these endpoints will be necessary to provide a more comprehensive understanding of the mechanisms underlying the effects of LIPUS and aerobic exercise rehabilitation. (4) Only male mice were included in the present study; therefore, potential sex-dependent differences in treatment responses remain unknown. (5) Only one LIPUS intensity and one aerobic exercise protocol were evaluated, and dose- or intensity-dependent effects could not be determined. (6) Cognitive function was not assessed, and the present behavioral findings should therefore be limited to locomotor and neurological outcomes.

A previous study has shown that pro-BDNF knockout leads to abnormal motor behaviors and early mortality in mice [37]. Moreover, although FNDC5/irisin has been recognized as a myokine that promotes metabolic health, its mechanistic pathways remain challenging to interpret due to limitations in detection and quantification methods, as well as the use of supraphysiological doses in experimental settings [38]. These issues represent important challenges that should be addressed in our future studies.

## 4. Materials and Methods

### 4.1. Animal Model of Ischemic Stroke

An MCAO model was used to induce focal cerebral ischemia in male mice as previously described [14]. Male C57BL/6 mice (6–8 weeks old) were divided into 7 groups (sham, sham + LIPUS, sham + exercise, MCAO, MCAO + LIPUS, MCAO + exercise, and MCAO + LIPUS + exercise; 9 mice/group). Mice were anesthetized. After cervical incision, the common carotid artery (CCA), external carotid artery (ECA), and internal carotid artery (ICA) were isolated and exposed. A 6–0 nylon monofilament was inserted through the CCA and advanced via the ICA to the origin of the middle cerebral artery (MCA), approximately 16–17 mm from the insertion point. The filament remained in place for 30–45 min to block blood flow. Wounds were sutured, and body temperature was maintained at 37 °C using an infrared heating lamp during recovery. Six animals underwent behavioral and histopathological/immunohistochemical assessments, while three animals underwent infarct area detection experiments.

Animals were randomly assigned to the experimental groups without restriction at the time of group allocation. Behavioral assessments were performed by an investigator blinded to group assignment. Because LIPUS stimulation and treadmill exercise require intervention-specific procedures, the personnel administering these treatments were necessarily aware of the group assignments and therefore were not blinded during intervention delivery.

This animal study was approved by the Institutional Animal Care and Use Committee (IACUC) of the College of Medicine, National Taiwan University (approval No. 20220320, date of approval: 1 February 2023). All animal experimental procedures were conducted in strict accordance with the guidelines for the care and use of laboratory animals established by the U.S. National Research Council.

### 4.2. Behavioral Assessments

(1) Open field locomotor activity: Spontaneous locomotor activity was assessed using an infrared photobeam detection system (Imetronic, Marcheprime, France). Mice were placed in a rectangular open-field arena (48 cm × 24 cm × 20 cm) divided into eight equal virtual zones. (2) Motor function evaluation: Neurological deficits were assessed using a modified Bederson [39] scoring system [40]. Evaluations were performed by an experimenter blinded to group assignment. Scoring criteria were as follows: 0: Normal; 1: Forelimb flexion; 2: Forelimb flexion with decreased resistance to lateral push; 3: Unidirectional circling; 4: Longitudinal spinning or severe loss of resistance; 5: No spontaneous movement. (3) Assessment of motor equilibrium performance on rotarod: Motor equilibrium performance was evaluated using the rotarod test [41]. Briefly, mice were pre-trained to acclimate to the apparatus prior to the experiment. During testing, each mouse underwent six consecutive trials on a rotarod rotating at a constant speed of 20 revolutions per minute (rpm). The latency to fall of a retention time was recorded, with a cutoff time of 180 s.

### 4.3. Exercise Rehabilitation Protocol

Aerobic treadmill training was performed to evaluate post-stroke rehabilitation effects. To minimize confounding effects, naïve mice underwent two weeks of pre-training (3 days/week, twice per day, 10 min each session). Running speed was set at 5 m/min during week 1 and increased to 10 m/min during week 2. Post-MCAO, treadmill training resumed on day 3. Mice initially ran at 5 m/min for 10 min, after which the intensity was gradually adjusted while maintaining a speed of approximately 10 m/min. These parameters were obtained from preliminary test results.

### 4.4. Ultrasound Stimulation

LIPUS, delivered by a 1.0 MHz ultrasound generator (ME740; Mettler Electronics, Anaheim, CA, USA), was administered as previously described [42]. The stimulation site was determined using a stereotaxic frame, with coordinates referenced to bregma according to a standard mouse brain atlas. The transducer was positioned 1.2 mm anterior and ±2.5 mm lateral to bregma, corresponding to the lateral motor cortex (M1/M2 region). LIPUS parameters were as follows: center frequency: 1.0 MHz; output power: 50%; exposure duration: 20 min; intensity (ISPTA): 100 mW/cm^2^. The pulse repetition frequency (PRF) was 100 Hz. The transducer had an effective radiating area (ERA) of 1 cm^2^ and produced a non-focused, collimated beam typical of therapeutic ultrasound systems. These parameters were obtained from preliminary test results. For three consecutive days before MCAO, LIPUS was applied daily immediately after a 10 min treadmill session. Post-reperfusion, following 2 days of recovery, mice received exercise rehabilitation or ultrasound therapy, or a combination of both.

### 4.5. Infarct Area Detection

Following neurological assessment, mice were euthanized and the brains were rapidly harvested. The brains were sectioned into 2 mm thick coronal slices and incubated in 2% 2,3,5–triphenyltetrazolium chloride (TTC) solution at 37 °C for 20 min, resulting in red staining, whereas nonviable regions remained unstained and were defined as infarct areas. Briefly, percentage hemisphere lesion volume (%HLV) = [total infarct volume − (volume of intact ipsilateral hemisphere − volume of intact contralateral hemisphere)]/contralateral hemisphere volume × 100%.

### 4.6. Histopathological Analysis (Hematoxylin–Eosin Staining)

Paraffin-embedded brain tissues were sectioned and deparaffinized in xylene, followed by a graded ethanol series (50%, 75%, and 90%). Tissue morphology was examined under 200×–400× magnification. Semi-quantitative scoring of pathological alterations in the brain, including neuronal necrosis and neuronal cell loss, hemorrhage, and gliosis with hemosiderin-laden macrophages, was assessed by a histopathologist in a blind test. The score was performed as follows: 0, no abnormality; 1+, sample change less than 25%; 2+, affecting 25–50% of the change; 3+, affecting 50–75% of the change; 4+, affecting more than 75% of the change.

### 4.7. Immunohistochemical (IHC) Staining Analysis

Sections of 4 μm thickness of paraffin-embedded brain tissue from the sham, MCAO, and MCAO combined treatment groups were incubated in a 60 °C dry oven before staining. After deparaffinization in xylene and rehydration through serial dilution of PBS/ethanol buffer solutions (100%, 75%, and 50%), antigen retrieval was performed by heating the sections in citrate buffer (pH 6.0). Endogenous peroxidase activity was blocked with 3% H_2_O_2_, and nonspecific binding sites were blocked by using 5% normal serum. The sections were incubated overnight at 4 °C with primary antibodies against BDNF (GeneText, Hsinchu City, Taiwan, GTX134514), FNDC5(BioAB, New Taipei City, Taiwan, A18170), or 8-OHdG (GeneText, GTX35250). After washing with saline buffer, the sections were incubated with the appropriate biotinylated secondary antibodies, followed by horseradish peroxidase-conjugated streptavidin. (BioGenex, Fremont, CA, USA) Immunoreactivity was visualized using 3,3′-diaminobenzidine as the chromogenic substrate, and the nuclei were counterstained with hematoxylin (Sigma-Aldrich, St. Louis, MO, USA). BDNF-, FNDC5-, and 8-OHdG-positive staining was quantified as the percentage of immunoreactive area using ImageJ software (NIH, Bethesda, MD, USA, Ver. 1.53k). At least five randomly selected, nonoverlapping fields from each section were analyzed by an investigator blinded to the experimental groups.

### 4.8. Protein Extraction and Western Blot Analysis

Protein concentration was quantified using the Bio–Rad protein assay. Equal amounts of protein were separated by 10% SDS–PAGE and transferred onto PVDF membranes. Successful transfer was verified by Ponceau S staining. Membranes were blocked in 0.5% Tween–20/5% non-fat milk/TBS for 1 h at room temperature and incubated overnight at 4 °C with primary antibodies for FNDC5 (Abcam, Waltham, MA, USA, ab174833), BDNF (GeneText, GTX134514), fibronectin (Santa Cruz, Dallas, TX, USA, sc–8422), catalase (Santa Cruz, sc–271803), SOD2 (Cell Signaling, Boston, MA, USA, #13141), and α–tubulin (Santa Cruz, sc–8035). After washing, HRP–conjugated secondary antibodies were applied for 1 h at room temperature. Signals were detected using enhanced chemiluminescence (ECL; Amersham, Lafayette, CO, USA). Densitometric quantification was performed using ImageJ.

### 4.9. Terminal Deoxynucleotidyl Transferase-Mediated dUTP Nick End-Labeling (TUNEL) Assay

Paraffin-embedded sections were deparaffinized and fixed in 4% paraformaldehyde for 30 min. After PBS washing, endogenous peroxidase activity was quenched using 3% H_2_O_2_. TUNEL reaction mixture (Promega, Madison, WI, USA) was applied, and slides were incubated at 37 °C in the dark for 1 h. Following PBS rinses, nuclei were counterstained with Hoechst 33258 for 15 min. Apoptotic cells were counted in 15 randomly selected fields under 200×–400× fluorescence microscopy.

### 4.10. Statistical Analysis

Data are presented as the mean ± SEM. Statistical analyses were performed using GraphPad Prism version 8.0 (GraphPad Software, San Diego, CA, USA) and analyzed by one-way analysis of variance (ANOVA), followed by Bonferroni’s post hoc multiple-comparisons test when the overall ANOVA indicated a significant difference. The number of animals analyzed in each group is indicated in the corresponding figure legends. All statistical tests were two-sided, and a *p*-value < 0.05 was considered statistically significant.

## 5. Conclusions

Taken together, our results suggest that LIPUS, aerobic exercise rehabilitation, and their combination provide protective benefits against post-stroke functional deficits and brain tissue injury under the experimental conditions in this study using a male mouse model with MCAO-induced ischemic stroke. The combined intervention produced greater effects than at least the effect of either treatment alone for selected outcomes, including greater increases in FNDC5 and BDNF protein expression in the brain of male MCAO mice. The outcomes of this preclinical study using a male mouse model may provide a basis for future translational research. Challenges remain to be addressed. Future studies may include evaluating additional exercise modalities, optimizing LIPUS parameters, and conducting more detailed molecular analyses, including pathway-specific inhibition or blockade studies to clarify the causal roles of the observed molecular changes. As a proof-of-concept study, the present work provides experimental evidence and preliminary molecular observations in male MCAO mice that support further investigation of LIPUS-based interventions combined with exercise rehabilitation for stroke recovery. However, because the LIPUS protocol included both preconditioning and post-stroke treatment, the independent contribution of each phase remains unclear. The underlying mechanisms and potential clinical benefits require further validation.

## Figures and Tables

**Figure 1:**
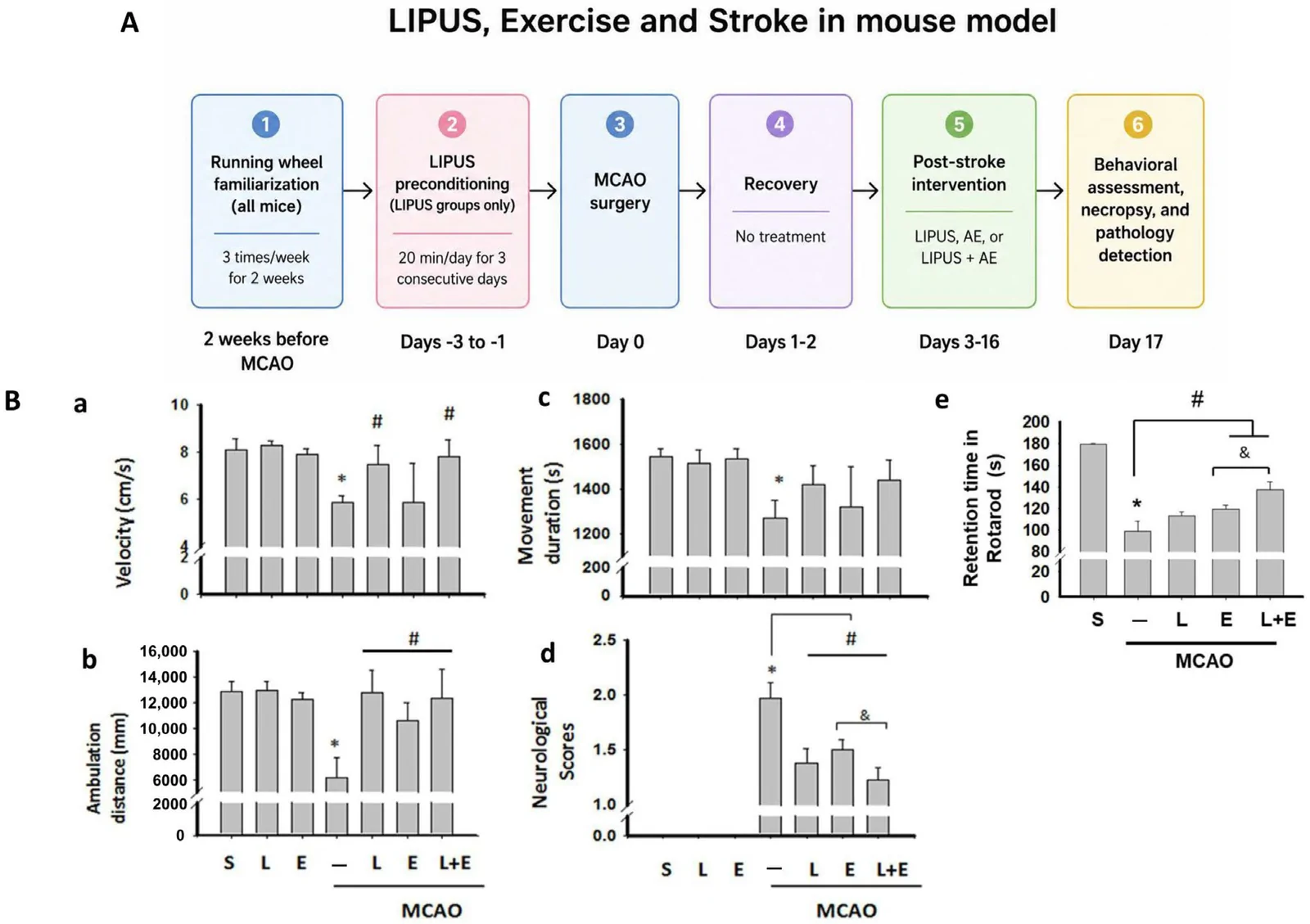
Experimental design and effects of LIPUS and aerobic exercise rehabilitation on locomotor and neurological outcomes following MCAO. (**A**) A workflow diagram for the MCAO mouse model with or without LIPUS or exercise treatment was shown. All mice underwent running-wheel familiarization three times per week for 2 weeks before MCAO. Mice assigned to the LIPUS-containing groups additionally received LIPUS preconditioning for 3 consecutive days (20 min/day) immediately before MCAO. Following MCAO surgery, mice were allowed to recover for 2 days. Beginning on postoperative day 3, mice received LIPUS, aerobic exercise (AE) rehabilitation, or their combination for 14 days. Behavioral assessments were performed 24 h after the final intervention. (**B**) Behavioral outcomes were evaluated by open-field locomotor activity tests (**a**–**c**), neurological deficit scoring (**d**), and rotarod performance (**e**). Data are presented as the mean ± SEM (*n* = 6 mice per group). (**a**) * *p* = 0.010, sham vs. MCAO; # *p* = 0.008, MCAO vs. MCAO + L; # *p* = 0.0052, MCAO vs. MCAO + E. (**b**) * *p* = 0.0001, sham vs. MCAO; # *p* = 0.0001, MCAO vs. MCAO + L; # *p* = 0.003, MCAO vs. MCAO + E; # *p* < 0.0001, MCAO vs. MCAO + L + E. (**c**) * *p* = 0.0002, sham vs. MCAO. (**d**) * *p* < 0.0001, sham vs. MCAO; # *p* < 0.0001, MCAO vs. MCAO + L; # *p* = 0.002, MCAO vs. MCAO + E; # *p* < 0.0001, MCAO vs. MCAO + L + E; & *p* = 0.013, MCAO + E vs. MCAO + L + E. (**e**) * *p* = 0.0001, sham vs. MCAO; # *p* = 0.019, MCAO vs. MCAO + E; # *p* = 0.002, MCAO vs. MCAO + L + E; & *p* = 0.048, MCAO + E vs. MCAO + L + E. S: sham; L: LIPUS; E: exercise.

**Figure 2:**
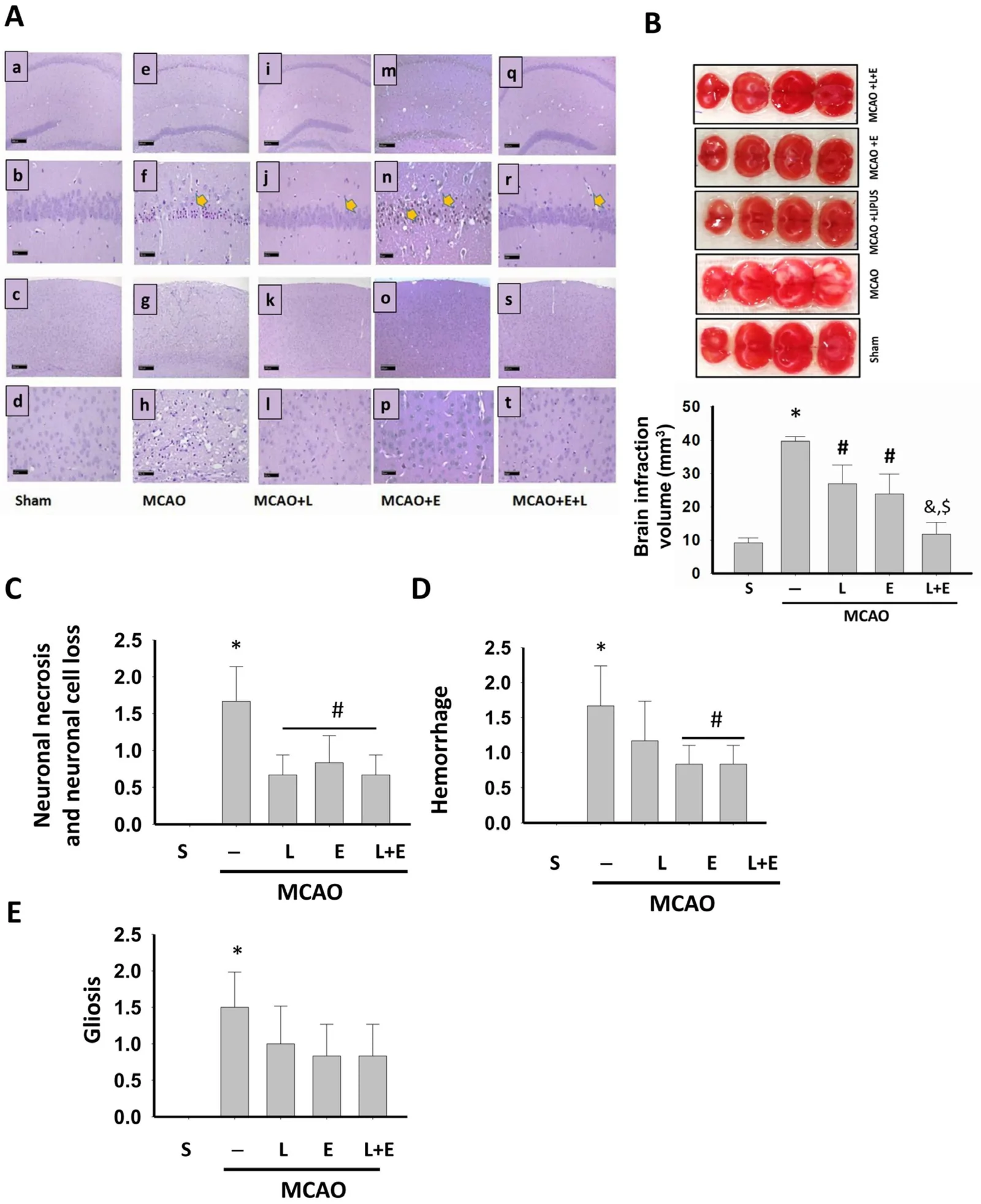
Effects of LIPUS and aerobic exercise on cerebral histopathological alterations following MCAO. Mice assigned to the LIPUS-containing groups received LIPUS for three consecutive days before MCAO induction, followed by LIPUS, aerobic exercise, or combined treatment for 14 days after MCAO. (**A**) Histopathological changes in brain tissue were detected by H&E staining. The histopathological changes in the regions of the hippocampus (**a**,**b**,**e**,**f**,**i**,**j**,**m**,**n**,**q**,**r**) and cerebral cortex (**c**,**d**,**g**,**h**,**k**,**l**,**o**,**p**,**s**,**t**) were shown in different groups. Scale bars: 200 μm in (**a**,**e**,**i**,**m**,**q**,**c**,**g**,**k**,**o**,**s**); 50 μm in (**b**,**f**,**j**,**n**,**r**,**d**,**h**,**l**,**p**,**t**). The arrows indicate areas that have altered. (**B**) Representative TTC-stained brain slices for infarct areas and the quantification were shown. (**C**–**E**) Semi-quantitative histopathological scores for neuronal necrosis and neuronal cell loss (**C**), hemorrhage (**D**), and gliosis (**E**). Data are presented as means ± SEM (*n* = 6 in (**A**,**C**–**E**); *n* = 3 in (**B**)). S: sham; L: LIPUS; E: exercise. (**B**) * *p* = 0.0001, sham vs. MCAO; # *p* = 0.0242, MCAO vs. MCAO + L; # *p* = 0.012, MCAO vs. MCAO + E; # *p* = 0.006, MCAO vs. MCAO + L + E; & *p* = 0.0315, MCAO + L vs. MCAO + L + E; $ *p* = 0.07, MCAO + E vs. MCAO + L + E. (**C**) * *p* = 0.003, sham vs. MCAO; # *p* = 0.0305, MCAO vs. MCAO + L; # *p* = 0.0476, MCAO vs. MCAO + E; # *p* = 0.0305, MCAO vs. MCAO + L + E. (**D**) * *p* < 0.0001, sham vs. MCAO; # *p* = 0.0177, MCAO vs. MCAO + E; # *p* = 0.0177, MCAO vs. MCAO + L + E. (**E**) * *p* = 0.0077, sham vs. MCAO. S, sham; L, LIPUS; E, aerobic exercise.

**Figure 3:**
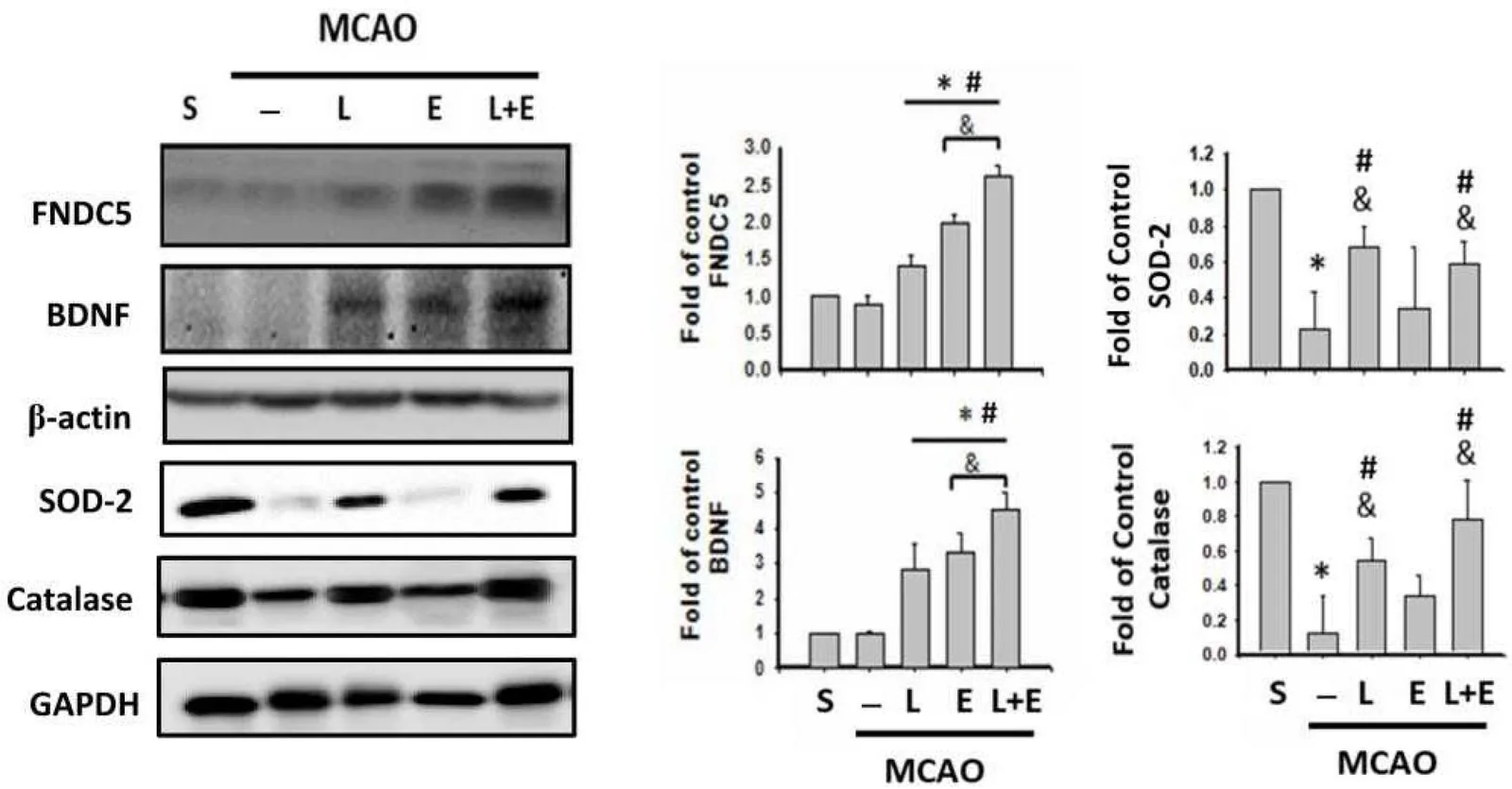
Effects of LIPUS and aerobic exercise on FNDC5, BDNF, SOD2, and catalase protein levels following MCAO. Using the experimental protocol shown in Figure 1A, mice received LIPUS, aerobic exercise (E), or their combination following MCAO. At the end of the treatment period, left cerebral hemisphere tissues were collected, and FNDC5, BDNF, SOD2, and catalase protein levels were determined by Western blotting. Representative immunoblots and densitometric analyses are shown. Data are presented as means ± SEM (*n* = 3). **FDNC5**: * *p* = 0.0101, sham vs. MCAO + L; * *p* < 0.0001, sham vs. MCAO + L + E, * *p* < 0.0001, sham vs. MCAO + L + E; *p* = 0.099, MCAO vs. MCAO + L; # *p* < 0.0001, MCAO vs. MCAO + E; # *p* < 0.0001, MCAO vs. MCAO + L + E; & *p* < 0.001, MCAO + L vs. MCAO + L + E, and & *p* = 0.009, MCAO + E vs. MCAO + L + E. **BDNF**: *p* = 0.0069, sham vs. MCAO + L; * *p* = 0.0029, sham vs. MCAO + E; * *p* < 0.001, sham vs. MCAO + L + E; *p* = 0.0068, MCAO vs. MCAO + L; # *p* = 0.0029, MCAO vs. MCAO + E; # *p* = 0.0015, MCAO vs. MCAO + L + E; *p* = 0.0083, MCAO + L vs. MCAO + L + E; & *p* = 0.0203, MCAO + E vs. MCAO + L + E. **SOD2**: * *p* < 0.001, sham vs. MCAO; # *p* = 0.013, MCAO vs. MCAO + L; # *p* = 0.018, MCAO vs. MCAO + L + E; & *p* = 0.043, MCAO + E vs. MCAO + L + E. Catalase: * *p* < 0.001, sham vs. MCAO; # *p* = 0.0061, MCAO vs. MCAO + L; # *p* < 0.001, MCAO vs. MCAO + L + E; & *p* = 0.0386, MCAO + L vs. MCAO + L + E.

**Figure 4:**
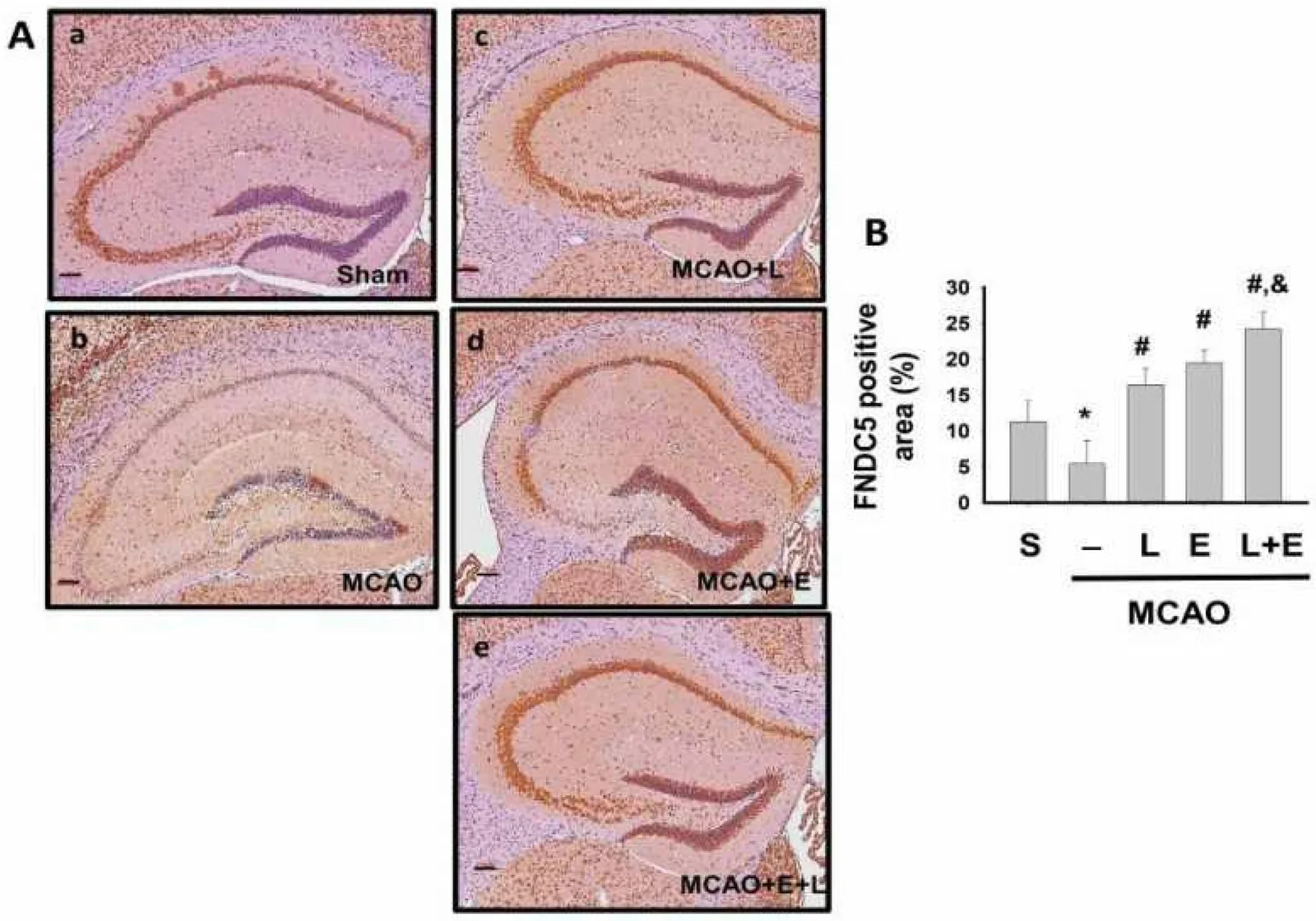
Effects of LIPUS and aerobic exercise on FNDC5 expression in the hippocampus of MCAO mice. Representative immunohistochemical staining images of FNDC5 in the hippocampus and dentate gyrus from the (**a**) sham, (**b**) MCAO, (**c**) MCAO plus LIPUS, (**d**) MCAO plus aerobic exercise, and (**e**) MCAO plus combined LIPUS and aerobic exercise groups in (**A**). Brown represents the region of the positive reaction. Quantitative analysis of the FNDC5-positive area is shown in (**B**). Data are presented as the mean ± SEM (*n* = 6). Scale bar = 100 μm. * *p* = 0.035, sham vs. MCAO; # *p* = 0.006, MCAO vs. MCAO + L; # *p* = 0.0013, MCAO vs. MCAO + E; # *p* = 0.0007, MCAO vs. MCAO + L + E; & *p* = 0.047, MCAO + L vs. MCAO + L + E. S: Sham; L: LIPUS; FNDC5: fibronectin type III domain-containing protein 5.

**Figure 5:**
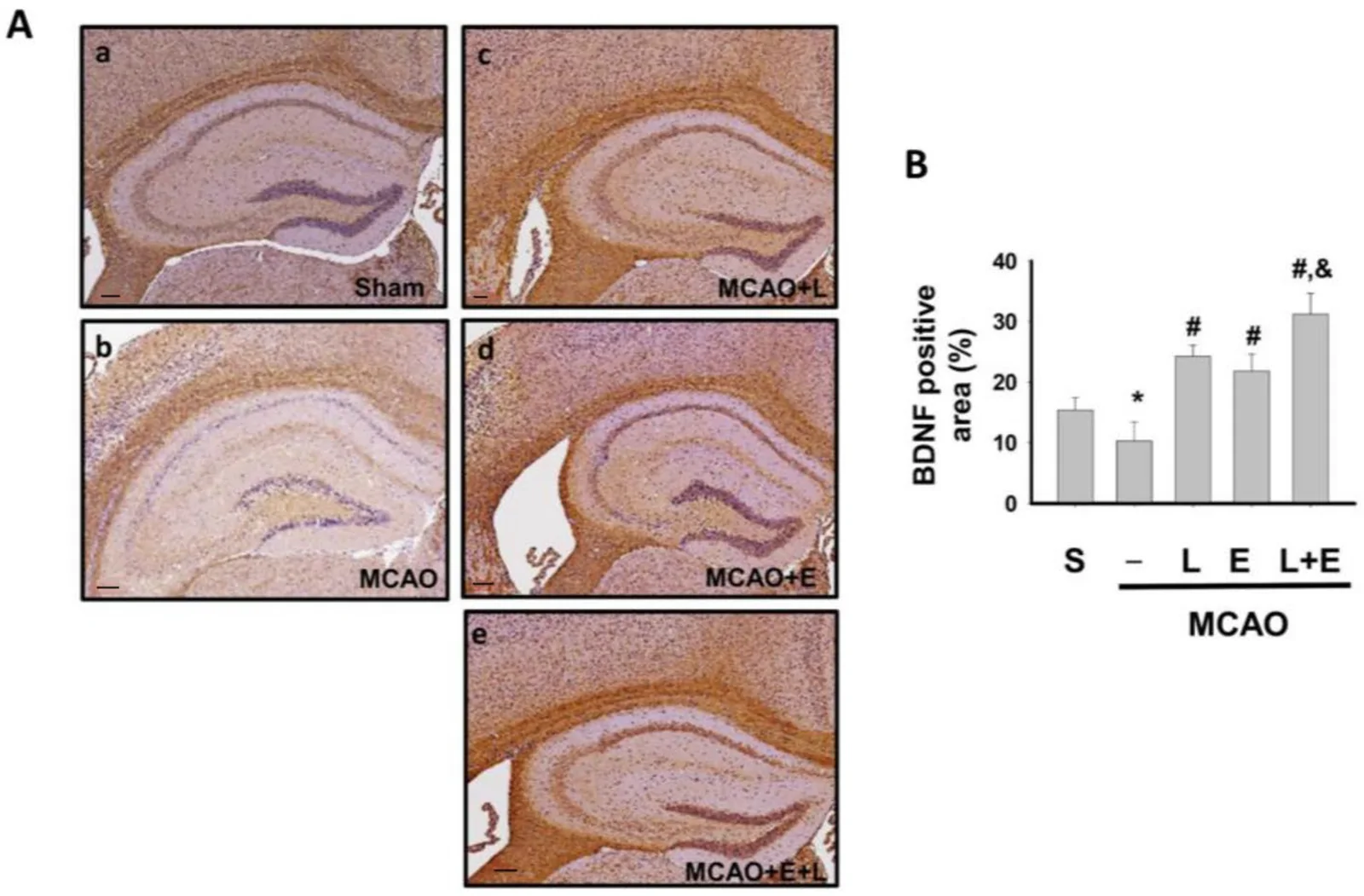
Effects of LIPUS and aerobic exercise on BDNF expression in the hippocampus of MCAO mice. Representative immunohistochemical staining images of BDNF in the hippocampus and dentate gyrus from the (**a**) sham, (**b**) MCAO, (**c**) MCAO plus LIPUS, (**d**) MCAO plus aerobic exercise, and (**e**) MCAO plus combined LIPUS and aerobic exercise groups in (**A**). Brown represents the region of the positive reaction. Quantitative analysis of the BDNF-positive area is shown in (**B**). Data are presented as the mean ± SEM (*n* = 6). Scale bar = 100 μm. * *p* = 0.0316, sham vs. MCAO; # *p* = 0.0017, MCAO vs. MCAO + L; # *p* = 0.0064, MCAO vs. MCAO + E; # *p* = 0.0001, MCAO vs. MCAO + L + E; & *p* = 0.029, MCAO + L vs. MCAO + L + E. S, sham; L, LIPUS; E, aerobic exercise; BDNF, brain-derived neurotrophic factor.

**Figure 6:**
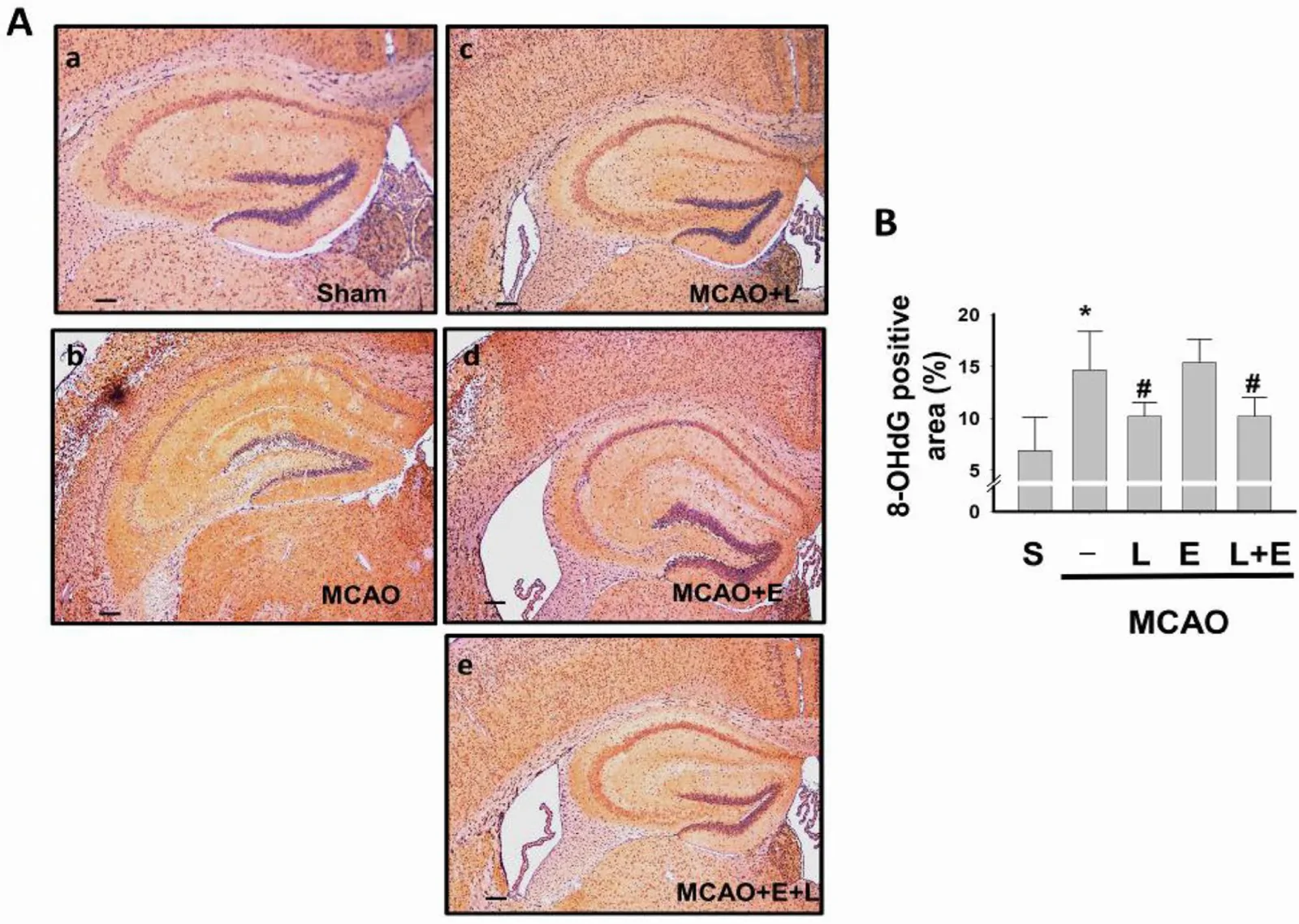
Effects of LIPUS and aerobic exercise on 8-OHdG expression in the hippocampus of MCAO mice. Representative immunohistochemical staining images of 8-OHdG in the hippocampus, including the dentate gyrus, are shown for the (**a**) sham, (**b**) MCAO, (**c**) MCAO + LIPUS, (**d**) MCAO + aerobic exercise, and (**e**) MCAO + LIPUS + aerobic exercise groups in (**A**). Brown represents the region of the positive reaction. Quantitative analysis of the 8-OHdG-positive area is shown in (**B**). Data are presented as the mean ± SEM (*n* = 6). Scale bar = 100 μm. * *p* = 0.02, sham vs. MCAO; # *p* = 0.0269, MCAO vs. MCAO + L; # *p* = 0.0475, MCAO vs. MCAO + L + E. S, sham; L, LIPUS; E, aerobic exercise; 8-OHdG, 8-hydroxy-2′-deoxyguanosine.

**Figure 7:**
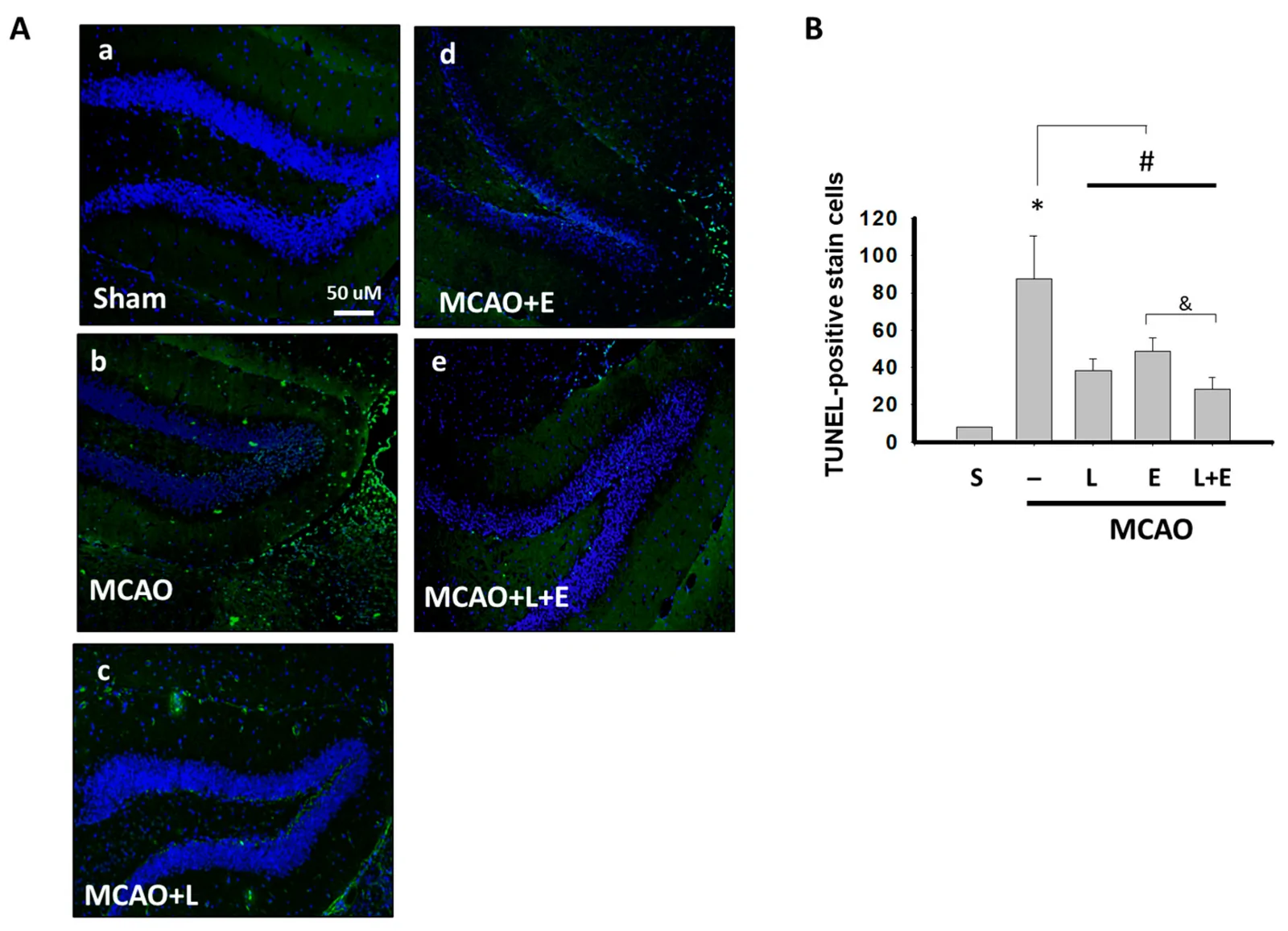
Effects of LIPUS and aerobic exercise on TUNEL positivity in the hippocampus of MCAO mice. Representative TUNEL-stained images of the hippocampus are shown in (**A**). (**a**) Sham; (**b**) MCAO; (**c**) MCAO + L; (**d**) MCAO + E; (**e**) MCAO + L + E. Green bright spots represent areas of positive reaction. Quantitative analysis of TUNEL-positive cells is shown in (**B**). Data are presented as the mean ± SEM (*n* = 6). * *p* < 0.001, sham vs. MCAO; # *p* < 0.001, MCAO vs. MCAO + L; # *p* < 0.001, MCAO vs. MCAO + E; # *p* < 0.001, MCAO vs. MCAO + L + E; & *p* = 0.0432, MCAO + E vs. MCAO + L + E. S, sham; L, LIPUS; E, aerobic exercise; TUNEL, terminal deoxynucleotidyl transferase dUTP nick-end labeling.

## Data Availability

The data presented in this study are available from the corresponding author upon reasonable request.
